# Anti-Inflammatory Diet for Women with Interstitial Cystitis/Bladder Pain Syndrome: The AID-IC Pilot Study

**DOI:** 10.3390/mps5030040

**Published:** 2022-05-18

**Authors:** Barbara Gordon, Cynthia Blanton, Rebekah Ramsey, Andrea Jeffery, Laura Richey, Rachel Hulse

**Affiliations:** 1 Department of Nutrition and Dietetics, Idaho State University, 1311 E Central Drive, Meridian, ID 83642, USA; andreajeffery@isu.edu (A.J.); laurarichey@isu.edu (L.R.); 2 Department of Nutrition and Dietetics, Idaho State University, 921 South 8th Avenue, Pocatello, ID 83209, USA; cynthiablanton@isu.edu (C.B.); rebekahramsay@isu.edu (R.R.); rachelhulse@isu.edu (R.H.)

**Keywords:** interstitial cystitis, bladder pain syndrome, anti-inflammatory diet, nutrition

## Abstract

Interstitial cystitis/bladder pain syndrome (IC/BPS) is a chronic condition characterized by pelvic pain coupled with urinary frequency and urgency. The underlying cause of IC/BPS is unknown; there is no cure. Dietary components exacerbate symptoms. The Anti-Inflammatory Diet for Interstitial Cystitis (AID-IC) employs a randomized, crossover design to evaluate the effect of a plant-based, low saturated fat diet on the quality of life of women with IC/BPS. Insights on the implementation of the protocol and reflections on the facilitators and barriers experienced during the pilot study follow. The logistics of the protocol proved time-consuming; however, the barriers were surmountable. Quantitative and qualitative findings suggest that the AID-IC therapeutic diet may have lessened symptoms and improved the quality of life for many of the women in the study.

## 1. Introduction

Interstitial cystitis/bladder pain syndrome (IC/BPS) is a chronic condition affecting more than 8 million women around the world [1,2]. Prevalence estimates vary greatly, ranging from 4.5 to 440 per 100,000 [3,4,5]. IC/BPS is characterized by pelvic pain, pressure, or discomfort coupled with daytime urinary frequency (10+) or urgency associated with pain, pressure, discomfort, and fear of wetting [6]. 

The underlying cause is unknown; there is no cure [6]. Presentation includes both periods of severe pain and lower urinary tract symptoms (flares) and periods of remission. Flares are described as mild to severe intensity. Patients report flare frequencies of daily to less than one per year [6,7] and durations of minutes to years [1,6,8]. When symptoms are severe, individuals may need to stay home from work or school, restrict socialization, and limit sexual encounters [8]. Women with IC/BPS also experience increased frequency of psycho-social trauma and suicidal tendencies [7]. Treatment may include a combination of medications, neurostimulation, physical therapy, and dietary modifications [8]. 

Chronic inflammation is a hallmark characteristic of IC/BPS [9,10]. Studies indicate that IC/BPS patients present with elevated blood and urine levels of proinflammatory cytokines. Jiang et al. examined serum samples from 30 IC/BPS patients and 26 controls and found significantly higher levels of C-reactive protein (CRP), interleukin (IL)-1B, IL-6, IL-8, and tumor necrosis factor-alpha (TNF-a) among those with IC/BPS [10]. Increased serum CRP levels in patients diagnosed with IC/BPS, compared with controls, were also reported by Chung and colleagues [11]. In a more recent study by Jiang et al., urine inflammatory markers were investigated for their IC/BPS diagnostic potential. This group reported significantly elevated urine levels of multiple cytokines in 127 IC/BPS patients versus 28 controls. Further, cytokine levels were significantly correlated with clinical characteristics, including self-reported symptom and pain scores [12]. 

For decades, individuals with IC/BPS have reported that the consumption of certain foods and beverages increases symptomology [13,14,15,16]. Clinical experience prompted the collection of commonly bothersome foods via a series of self-report surveys [13,14,15,16,17,18]. In 2014, Shorter et al. published a validated survey yielding a list of foods and beverages that the vast majority (90%) of women with IC/BPS appear to find bothersome [18]. Items include coffee and tea (caffeinated and decaffeinated), soda, alcohol, citrus juices and fruits, cranberry juice, tomatoes and tomato products, hot peppers, spicy foods, and foods and beverages containing artificial sweeteners [18]. There are also individual variations in the food/beverage sensitivities [18]. Given these findings, dietary modifications were included in clinical guidelines for six medical societies across the globe [6,19,20,21,22,23].

The pathophysiology underlying the association between symptoms and dietary components is not well understood in IC/BPS. One theory is that damage to the bladder urothelial barrier allows urinary solutes to invade the urothelium and produce noxious stimuli that provoke IC/BPS symptoms [24]. Others hypothesize that consumption of foods that irritate the gastrointestinal tract may stimulate nerves between the GI tract and the bladder; thereby, “neural cross-talk” produces bladder discomfort [25]. Nazif et al. purport that peripheral or central neural upregulation underlies the pathology of IC/BPS; specifically, the release of neurogenic inflammation from sensory nerves may be a catalyst for the pain and urinary symptoms of this condition [26]. Elliott et al. discovered that artificial sweeteners activate T1R2/3 sweet taste receptors expressed in the bladder (human and rat), causing bladder smooth muscle contractions [27]. This may explain the sensitivity to artificial sweeteners reported by those with IC/BPS.

The potential role of diet as a contributing factor to diseases associated with chronic systemic inflammation has been suggested [28,29,30]. Shivappa et al. created the Dietary Inflammatory Index (DII), which rates the influence of foods, dietary components, or nutrients based on their anti-inflammatory property. The degree of inflammation was ranked by the magnitude of the effect of an item on six inflammatory biomarkers (IL-1β, IL-4, IL-6, IL-10, TNF-α, and C-reactive protein) [29]. Forty-five items were assigned DII scores on a scale of −1 to +1 (−1 = strongly anti-inflammatory, 0 = no effect, and +1 = strongly proinflammatory) [29]. A meta-analysis concluded that a high DII score is likely associated with the harmful inflammatory effects related to a variety of health outcomes [31]. The developers of the D-II refined the tool by including an energy adjustment calculation in the algorithm; the E-DII is the preferred tool for assessing the influence of dietary choices on chronic systemic inflammation [32].

Eating patterns that are high in saturated fat, cholesterol, protein, sugar, and salt coupled with regular consumption of ultra-processed foods are associated with a higher prevalence of chronic diseases with inflammatory components [28]. Studies suggest that dietary intake of saturated fat provokes inflammation by activating the Toll-like receptor (TLR)-4 cell-signaling pathways [33,34]. In a molecular dynamics study, it was found that the saturated fat palmitic acid (found in palm oil) produced TLR-4-induced changes to metabolism on a cellular level, as well as alterations in gene expression, lipid metabolic pathways, and membrane lipid composition. The authors purport this contributes to the inflammation related to high consumption of dietary saturated fats [35]. Oberbach et al. reported that saturated fatty acids produced considerable changes in bladder smooth muscle cells. Specifically, bladder inflammation and vascular changes in association with endothelial dysfunction were observed [36]. The authors suggested that these changes may be responsible for clinical symptoms of IC/BPS. Other researchers noted a relationship between the ratio of saturated to polyunsaturated intake and lower urinary tract symptoms [30,37]. Of note, Maserejian et al. reported that modifying the dietary intake of the ratio of saturated fat to polyunsaturated fat may help control urinary urgency and frequency in women [37]. A potential link between diets high in saturated fat and IC/BPS symptomology emerges [30,33,34,35,36].

The goal of the AID-IC pilot study was to investigate the efficacy of an anti-inflammatory diet as a complementary treatment for pharmacological therapies to manage symptom severity and, thereby, contribute to improved quality of life. To date, no studies have been conducted investigating the feasibility of diminishing IC/BPS symptom severity by influencing inflammatory activity; specifically, controlling lipid intake and increasing antioxidant intake. An overview of the design and findings of a pilot study conducted with women with IC/BPS is detailed below. Facilitators and barriers that emerged during the pilot are discussed and revisions to the study protocol are offered.

## 2. Materials and Methods

The AID-IC pilot study employed a mixed-methods, crossover design to evaluate the effect of a plant-based, low saturated fat diet on IC/BPS symptom mitigation. It was implemented across 11 months, January through November 2021. The AID-IC study employed an adapted version of the randomized, cross-over Anti-inflammatory Diet In Rheumatoid Arthritis (ADIRA) protocol, which investigated if an anti-inflammatory diet diminished rheumatoid arthritis symptoms and improved quality of life [38].

### 2.1. Development of AID-IC Therapeutic and Control Diets

#### 2.1.1. AID-IC Therapeutic Diet

The AID-IC therapeutic diet incorporated specific foods found to be beneficial in controlling inflammation, while eliminating foods thought to increase inflammation. The E-DII was used to identify items with anti-inflammatory properties, such as foods rich in provitamin A carotenoids, vitamins C, D, and E, and certain spices (garlic, ginger, oregano, rosemary, and thyme) [29,32]. Foods and beverages identified by Shorter et al. as commonly bothersome foods for those with IC/BPS were restricted; for example, citrus foods, tomatoes and tomato products, and alcohol [18].

A typical plate included two ounces of protein, half a plate of vegetables, and a quarter plate of whole grains. Snacks consisted of fresh fruits. The only fat used in the prepared meals was olive oil. About 28% of kilocalories came from animal foods and none of the prepared meals included red meat. The AID-IC diet included cruciferous vegetables, low-fat dairy products, whole grains, nuts and berries, oily fish (3–4 times per week), and meatless entrees (1–2 times per week). Each entree provided about 500 kilocalories and one third of the RDAs for micronutrients. To confirm nutritional composition, meals were entered into a nutrition analysis software (EshaTrak, Salem, OR, USA) and modifications were made, as needed. 

The research team contracted with a commercial service for meal production and delivery (MealPro, Sacramento, CA, USA). The prepared meals were provided for lunch and dinner on the five weekdays (one-week cycle menu). The portion sizes were pre-established and the meals pre-cooked. Each meal was plated in a vacuum-sealed, microwave/oven-safe container. The packaging allowed for meals to be stacked for cold storage. Two weeks of meals were delivered in thermal boxes on dry ice.

#### 2.1.2. Control Diet

The control diet was the standard nutrition counseling protocol for those with IC/BPS, specifically the restriction of both individual and commonly bothersome IC/BPS. Registered dietitian nutritionists (RDNs) employed the evidence-based nutrition care process, which includes crafting a nutrition intervention based on the findings of a nutrition assessment. During the assessment, among other things, kilocalories required to meet nutritional needs and maintain current weight were calculated. Nutritional needs related to other health conditions were combined with IC/BPS recommendations. Due to budgetary limitations of the pilot study, participants were responsible for providing their own meals during the control diet phase of the study.

### 2.2. Recruitment and Screening

Enrollment was conducted from January to February 2021. A convenience sample of women (18 years or older) with physician-diagnosed IC/BPS was referred by the Idaho Urologic Institute (IUI, Meridian, ID, USA), a specialty medical practice. Given that food sensitivities affect nearly all women with IC/BPS (compared to less than half of men with the condition) the protocol only included women. In addition, women were excluded if IC/BPS symptoms currently demanded active medical attention. Other exclusion criteria included being intolerant, allergic, or unwilling to consume the food items in the AID-IC protocol, life-threatening diseases, pregnancy, or lactation (see Table 1). 

### 2.3. Intervention

Participants were assigned study IDs, which were sequentially entered into an online randomizer (gigacalculator.com, accessed on 15 February 2021). Group 1 received the AID-IC diet for 10 weeks, while Group 2 was assigned to the control diet. After a two-week washout period, Group 1 received the control diet and Group 2 the AID-IC diet. All of the participants were instructed to go back to their usual diet during the two-week washout period. 

Participants were assigned to one of three RDNs, and they met with the dietitian four times during the study. At the first visit, the RDNs reviewed the study details, obtained written consent, and performed a basic medical history. In addition, during visits one and three, the dietitians reviewed study protocols (therapeutic or control diet), advised participants to restrict trigger foods, computed kilocalories needed to maintain body weight, and provided nutrition counseling. Participants were provided printed and video versions of educational materials for the control or test diet (meal plans, recipes, and other information in plain language). At all visits, the RDNs walked participants through the dietary recall and disease activity surveys. 

Adherence was monitored via periodic correspondence, either in person or telephonically, with the RDN. Participants were encouraged to report any side effects to both the RDN and their IUI health care provider. Table 2 provides an overview of enrollment and data collection tasks stratified by study time points per SPIRIT requirements.

### 2.4. Data Collection

#### 2.4.1. Health and Dietary Information

A basic health history was collected by the RDNs, including comorbid conditions, prescription and over-the-counter medications, supplements, IC/BPS trigger foods, height, weight, and activity level. Body mass index (BMI) was calculated. This data was entered into an online survey created in Qualtrics XM (Provo, UT, USA). Using the Mifflin-St Jeor equation, daily kilocalorie needs were calculated for each individual. In addition, dietary intake data for 24-h recalls (one weekday and one weekend day) were collected using the Automated Self-Administered 24-h (ASA24) Dietary Assessment Tool, version 2018, developed by the National Cancer Institute, Bethesda, MD, USA (https://epi.grants.cancer.gov/asa24/respondent/citations.html, accessed on 13 November 2020). 

#### 2.4.2. Disease Activity

Three validated tools were employed to collect participant data on the degree of pain, urinary symptoms, sexual dysfunction, and quality of life—the 9-item Genitourinary Pain Index (GUPI, degree of pain and urinary symptoms), 19-item Female Sexual Functioning Inventory (FSFI, pain and frequency during and after intercourse), and 6-item RAND Interstitial Cystitis Epidemiology (RICE) Bladder Symptom Impact Scale (quality of life) [39,40,41]. The primary outcome data (beneficial changes in quality of life) were collected via the three surveys. The surveys were built on the Qualtrics XM platform and administered by the RDNs.

Blood samples were collected to evaluate inflammatory markers—one of the secondary outcomes of the study—four times, both pre/post-therapeutic diet and pre/post-control diet. Tests for three biomarkers were run, including tumor necrosis factor (TNF)-α, interleukin (IL)-1, and C-reactive protein. Given the location of the study, a contract phlebotomist was hired to draw and pack the samples, which were then transported by a courier service to a reference lab for development.

#### 2.4.3. Palatability and Acceptability of AID-IC Therapeutic Diet

A 14-question survey, employing a 5-point Likert rating scale, was developed to assess the palatability and ease of adoption of the AID-IC therapeutic diet. A link to the survey was sent out through secure email five weeks into the first 10-week study period to all of the participants consuming the study diet. The participants were given one week to complete the survey. Reminder emails were sent as needed. The survey was adapted from a palatability and feasibility questionnaire used to assess a Paleolithic diet among a group of Australian women [42]. The individual palatability of each of the 10 AID-IC therapeutic meals was rated from very unpleasant to very tasty. One question rated the overall satiety that the diet provided, ranging from very full and satisfied to very hungry and dissatisfied. Two questions focused on adaptation of the study diet; responses ranged from very easy to very difficult. There was one question rating the ease in coping with social situations while eating the study diet, ranging from very easy to very hard. The survey was also built in Qualtrics. 

#### 2.4.4. Participant Experience

Upon completion of the nutrition intervention, online focus groups were conducted to collect qualitative data on the participant experience. The 1 1/2-h focus group was conducted via Zoom and facilitated by one of the RDNs. A moderator’s guide was developed with questions on five topics: (1) meals (delivered and self-prepared) and adherence, (2) IC/BPS symptoms (flares, pain, urgency/frequency, and need for medical appointments), (3) quality of life (intimacy, activities of daily living, and activity level), (4) things learned (triggers, general eating patterns, and other), and (5) recommendations and miscellaneous comments. A USD 50 Amazon gift card was offered as an incentive. Transcripts were downloaded and personal identifying information removed. 

### 2.5. Data Analysis

SPSS v. 27 (IBM, Chicago, IL, USA) was employed to conduct statistical analysis. Given the small sample size, normality was analyzed using the Shapiro–Wilk test. Descriptive statistics were calculated. T-tests were employed to calculate significant differences in dietary intakes pre/post-control diet and pre/post-test. ANOVAs were used to evaluate significant relationships between changes in disease activity (symptomatology, pain, and quality of life) and pre/post-therapeutic diet and pre/post-control diet. Rows containing missing data were excluded from the analysis. E-DII scores (using 28/45 items) for each participant’s pre/post-intervention were calculated using the protocol detailed by the authors of the tool [29,32]. Focus group findings were narratively compiled.

## 3. Results

### 3.1. Participant Flow and Demographics

Across two months, 37 women were recruited; 35.48% were excluded because of dietary restrictions (gluten-free, does not eat carbohydrates, or only eats organic foods), demands of the study protocol (in-person blood draws and distance to the facility), and recently learning of pregnancy. Of the remaining 26 women, 8 did not respond to appointment scheduling requests or did not show for initial screening appointments. The remaining 18 participants were randomized; six of them dropped out during the screening process (demands of protocol, out of town travel, or desire to lose weight). 

**Figure 1:**
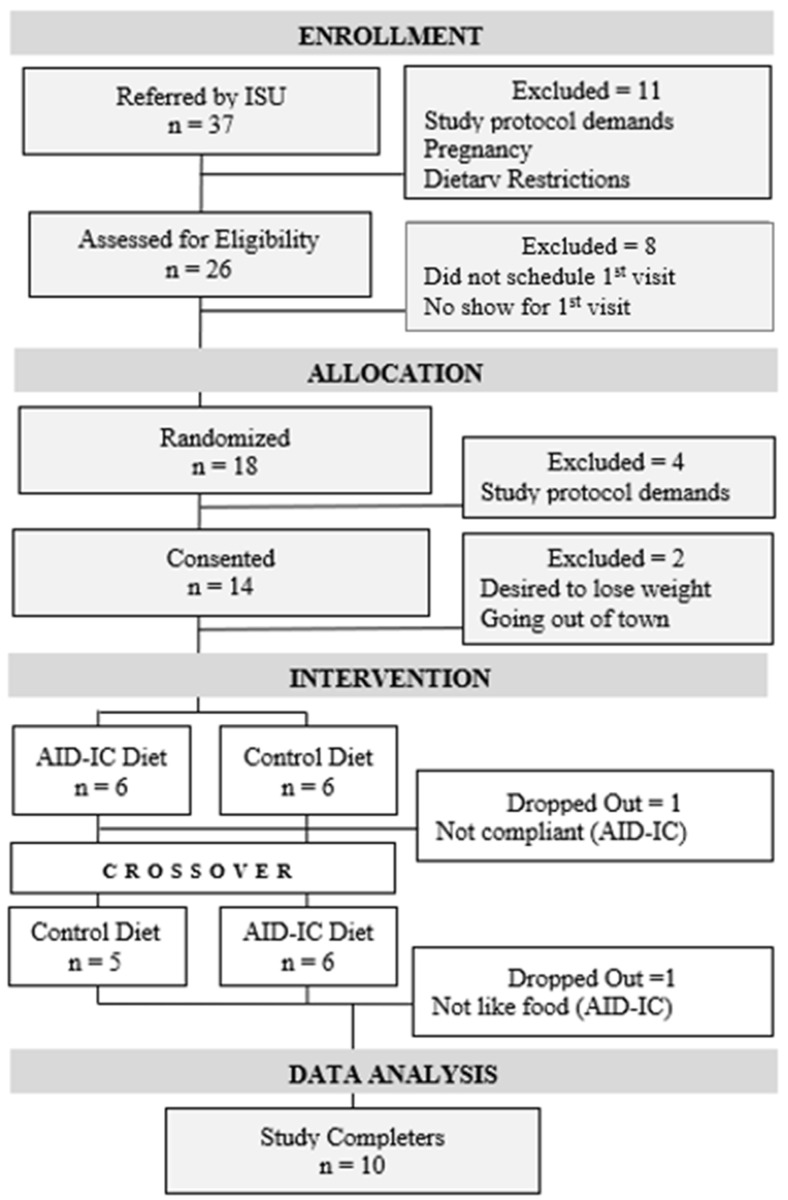
Participant flow chart (CONSORT).

Twelve women took part in the pilot study; 10 completed the intervention (83.33%). One reported that adherence to the therapeutic diet protocol was challenging (first 10 weeks); the other found the prepared meals unpalatable (second 10 weeks). None of the participants reported adverse reactions (see Figure 1).

All of the participants identified as White/Non-Hispanic; they were primarily over 45 years, with incomes above USD 50,000 (8/10, 80%). Most of the women were overweight/obese (8/10, 80%). Comorbid pain conditions (chronic headache, fibromyalgia, and vulvodynia) were reported by 70% of the cohort. The most common IC/BPS trigger foods were tomatoes/tomato products—60% reported these items as bothersome. Half of the participants also identified alcohol and spicy foods as bothersome, and 30% citrus food and coffee (see Table 3).

### 3.2. Nutrient Composition Therapeutic vs. Control Diets

Table 4 provides descriptive statistics for the E-DII scores and therapeutic/control diets. The dataset met the test of normality, Shapiro–Wilk test < 0.01. Comparison of means of the nutrient composition of the control vs. therapeutic diets yielded only one significant change in intake. Vitamin C intake significantly decreased while participants were on the test diet (*p* = 0.02). No significant associations emerged for intakes pre-/post-control diet.

Testing effect of timepoint (control vs. therapeutic diet) was conducted on ASA24 dietary intake data. When participants were on the therapeutic diet, intakes of solid fats (*p* ≤ 0.01), saturated fat (*p* = 0.02), and refined grains (*p* ≤ 0.01) significantly decreased, and seafood (*p* ≤ 0.01) and vitamin B12 (*p* = 0.03) significantly increased. There were no significant differences for intakes of other macro- and micro-nutrients. Findings were confirmed with the Tukey’s test. Calculations for individual participant found significant differences in nutrient intakes on therapeutic vs. control diets for three women. The off-study diets of the other participants were, thus, not significantly different from the therapeutic diet.

The E-DII scores ranged from −4.33 to 3.43 anti-inflammatory to proinflammatory, respectively. The mean E-DII score for participants on the AID-IC therapeutic diet was −1.50 (anti-inflammatory) and, for the control diet, was −0.98, which is also anti-inflammatory. The mean E-DII scores for the pre/post-test diets were significantly different (*p* = 0.05); however, no significant difference emerged between the pre/post-control diets. A simple linear regression was calculated to predict if the E-DII score was associated with the therapeutic diet (test vs. control diet). No significant association emerged. 

Mean ratings were calculated for the responses to the acceptable and palatability survey. One participant selected the same rating for all of the questions. These outlier scores were not included in the analysis. The mean rating for one meal was tasty to very tasty (1.80 ± 0.93); of note, it was a meatless entrée. Seven meals were rated okay to tasty (mean ranges: 2.33 ± 1.23 to 2.8 ± 1.23). The two salmon meals ranked okay to a little unpleasant (3.00 ± 1.25, 3.20 ± 1.23). Means for the four acceptance factors were computed; they were satiety (1.78 ± 0.83, very full and satisfied), difficulty eating this way (2.11 ± 0.78, easy), difficulty adapting to eating plan (2.67 ± 0.87, easy to neutral), and difficulty eating this way coping with social situations (2.89 ± 0.78, easy to neutral).

### 3.3. Impact of Diet on Disease Activity

Both mean RICE and GUPI scores for those in the treatment group were lower than for those in the control group (11.33 vs. 12.56 and 5.00 vs. 4.50, respectively). The inverse was true for the FSFI scores; the treatment group scores were higher than those in the control group (5.0 vs. 4.5, respectively). A series of one-way between-subjects ANOVAs were conducted to compare the effect of the test and control diets on disease activity (scores of the three surveys administered pre/post-intervention vs. pre/post-control diet). The FSFI dataset met the test of normality, Shapiro–Wilk test (<0.03). Though not significant for the RICE and GUPI datasets, the Shapiro–Wilk statistic was below 1.96, which is sufficient to establish normality. Significant changes were found for three of the five factors evaluated in the RICE Bladder Symptom index. Interest in life (*p* < 0.01), social life (*p* = 0.04), and ability to fulfill home responsibilities (*p* = 0.01) significantly improved; the overall RICE score was also significant (*p* = 0.05). Differences in energy level and mood were not significant. Genitourinary Pain Index scores were significantly less for pain in the pubic or bladder area (*p* = 0.01), pain/burning during voiding (*p* = 0.01), and discomfort during/after intercourse (*p* = 0.01); however, the total GUPI subscores and overall score was not significant. Significant improvements for the Sexual Functioning Inventory scores included sexual desire/interest (*p* = 0.01), confidence that will become aroused (*p* = 0.01), and ability to maintain lubrication (*p* = 0.04). Table 5 provides descriptive statistics and results of paired T-tests for the totaled survey scores.

Four viable blood samples were analyzed for significant changes in three inflammatory biomarkers—TNF-alpha, IL-B, and C-reactive protein. ANOVAs failed to calculate because of the small dataset. Improper handling by the courier service resulted in tainted samples and insufficient data for the biomarkers. This incident was reported to the Institutional Review Board. 

### 3.4. Patient Experience

A total of 10 out of the 12 women participated in the focus groups. The AID-IC therapeutic diet lessened IC/BPS symptoms and improved quality of life for 70% of the focus group participants. Newly diagnosed women appeared to find the intervention more beneficial. Participants also noted that the AID-IC diet served as an elimination diet; when they returned to preintervention eating patterns, they were able to identify individual trigger foods. Some participants said that, postintervention, they continued to follow the AID-IC diet, using the patient education materials as a guide. 

Participants found the meals very ample. Some consumed leftover portions of the delivered meals on the weekends. Others shared that, due to the satiety of the meals, they did not desire between-meal snacks. Concerns included the mushiness of the vegetables and lack of variety (consumption of the same menu items every week for 10 weeks). 

## 4. Discussion

Despite dietary modifications being considered standard care for this population [6,19,20,21,22,23], there are only three published clinical trials that have investigated the efficacy of dietary changes for helping to manage IC/BPS symptoms [43,44,45]. A pilot study (*n* = 25) conducted by Hanley et al. included dietary modification as part of a multimodal intervention (behavioral, pharmacologic, and endoscopic) [43]. In addition to fluid restrictions, participants restricted acidic foods and those high in arylalkylamines. The researchers, however, reported no discrete findings on the influence of the dietary modifications [43]. A double-blind, placebo-controlled trial (*n* = 30) evaluated the influence of caffeine on the exacerbation of IC/BPS symptomology. The intervention group consumed a caffeine pill (equivalent to one cup of coffee), the control group a placebo. No significant difference in pain or voiding volume was found between the two groups [44]. Oh-oka reported on the findings of a small (*n* = 30), 1-year clinical trial employing an intensive systematic dietary manipulation (restriction of commonly bothersome foods for individuals with IC/BPS). Statistically significant improvements (*p* < 0.05) were reported for all evaluated factors after three months and throughout the final nine months of the trial [45]. None of the above studies have been replicated. 

The lack of clinical trials evaluating dietary adaptations for those with IC/BPS was a facilitator. Many participants expressed their appreciation to the researchers for conducting the study. The ADIRA model [38] of providing home-delivered meals also encouraged adherence; focus group participants spoke positively about the nominal meal preparation and ease of reheating the meals. 

Both the testing effect of timepoint and DII score analyses demonstrate that the therapeutic diet achieved the goal of controlling for lipid intake and promoting the intake of anti-inflammatory nutrients and dietary components. Of note, the E-DII score range (4.33 to 3.43) was within the expected range for a dataset of 29 items; per Hébert et al., for 25–30 items, the expected range is from −5.5 to +5.5 [32]. More than 300 studies have been published verifying a relationship, albeit not causal, between the E-DII score and chronic systemic inflammation.

The disease activity scores for the RICE and GUPI (both subscales and overall) were indicative of lesser IC/BPS symptomatology for those on the therapeutic diet compared with the control diet. Scores for the FSFI, however, revealed an inverse relationship between the anti-inflammatory diet and sexual functioning scores. This may reflect the lack of power in the sample size and/or the influence of symptoms to confounders, e.g., stress and comorbid conditions. 

The small sample size of this pilot study introduced the risk of a type II error. A power calculation can help address this weakness. Given the study design (two independent groups, binomial endpoint, alpha = 0.05), for an incidence of 50%, 116 participants are required. Recruitment from a single site also weakens the generalizability of the findings. The participant pool lacked racial/ethnic and socioeconomic diversity. Lebowitz et al. note that multi-site trials are more apt to yield heterogeneous participant samples. In addition, these authors emphasize the need for large, multi-site trials when evaluating optimal treatments among individuals with chronic conditions [46]. 

Based on the findings of this pilot study, the AID-IC protocol offers a feasible approach for assessing the impact of an anti-inflammatory diet among community-dwelling individuals with IC/BPS. Significant beneficial changes were found on the three disease activity surveys. Qualitative findings from focus group participants support these quantitative findings.

### Strengths and Limitations

The utilization of RDNs was advantageous because of their training in both clinical care and program management. Furthermore, the strategy of having each RDN follow the same participants throughout the trial helped to establish a rapport, which has been found to be a benefit for urogynecological trials. Analysis of interviews with women considering enrolling in a study of uterine prolapse treatment (*n* = 8), for example, emphasized the importance of establishing rapport and building a positive relationship with the trial participants [47].

COVID-19 requirements during the study period included the option for telenutrition or in-person visits. Virtual visits were not a barrier to establishing rapport with the participants; others reported similar experiences with conducting clinical trials during COVID-19 restrictions [48]. The initial time allotted of one hour per data collection visit, however, was insufficient. Given the scope of the data collection efforts, most visits required 1 1/2-h per participant. 

The prepared meals added an element of scientific control to the community-dwelling setting of this study; however, delivery issues emerged during the study. The first delivery date coincided with a winter snowstorm. Shipments were delayed due to weather; some meals arrived thawed and replacement meals had to be sent out. In addition, during the 10-week phases, many of the participants traveled. This required co-ordinating partial or full deliveries to alternative locations. These expenses had not been calculated into the study budget. 

Menu procedural issues also emerged. The one-week cycle menu did not optimally mirror the dietary diversity that participants consumed before the trial. The Clemson University Cooperative Extension Service advocates for at least a 2-week cycle menu for community-dwelling individuals [49]. Other resources recommend at least a 4-week cycle menu to offer a varied diet desired by most individuals [50]. Moreover, of note, the frozen vegetables were described by focus group participants as “too mushy.” In a review article on frozen vegetable production, van der Sman provides eight recommendations, such as employing a “high temperature short time” blanching procedure to help prevent degradation of cell walls and maintain the texture of the vegetables [51]. The two salmon meals received the lowest taste ratings, perhaps because, in the state of Idaho (United States), freshly caught salmon is very accessible. The flash-frozen meal may have not been the same quality that participants typically consume. 

The AID-IC protocol employed a two-week washout period compared with the three months used in the ADIRA study. Other dietary interventions have found promising outcomes with shorter washout periods. A clinical trial evaluating the efficacy of the elimination diet for women with migraines and irritable bowel syndrome found that a three-week washout period was adequate to produce measurable outcomes [52]. A longer washout period may have produced more significant differences between the therapeutic and control diet phases for the study outcome variables.

## 5. Conclusions

The AID-IC protocol offers a feasible model for evaluating dietary modifications for IC/BPS patients. Analysis of disease activity surveys and focus group findings demonstrate that the protocol lessened IC/BPS symptoms and improved the quality of life for many of the women in this study. A larger and more diverse sample is required to mitigate the risk of type II error and increase the generalizability of the findings. Improvements in menu design and meal production are also needed to optimize the impact of an anti-inflammatory diet.

## Figures and Tables

**Table 1:** Inclusion and exclusion criteria for AID-IC pilot study.

Inclusion Criteria	Exclusion Criteria
Women with physician diagnosis of IC/BPS 18 years or older Foods/beverages trigger IC/BPS symptoms	Males with IC/BPS Women with Hunner’s lesions IC/BPS currently demanding active medical attention Life-threatening diseases Pregnant or lactating Irritable bowel syndrome, gluten intolerance/sensitivity, lactose intolerance Vegetarian or vegan Intolerant, allergic, or unwilling to consume AID-IC diet Life-threatening diseases

**Table 2:** Enrollment and data collection stratified by study time points (SPIRIT requirements).

	Enrollment	Allocation	Intervention				
Time Point	Pre-Intervention		Start 1st 10 Weeks	End 1st 10 Weeks	Wash Out 2 Weeks	Start 2nd 10 Weeks	End 2nd 10 Weeks
ENROLLMENT							
Eligibility screen	X						
Randomization		X					
Information consent		X					
INTERVENTION							
AID IC then Control			X	X		X	X
Control then AID IC			X	X		X	X
ASSESSMENTS							
ASA24			X	X		X	X
Kilocalorie requirements			X			X	
Primary outcomes (quality of life) *							
GUPI			X	X		X	X
FSFI			X	X		X	X
RICE			X	X		X	X
Secondary outcomes (inflammatory markers, acceptability of therapeutic diet)							
Tumor Necrosis Factor-alpha **			X	X		X	X
Interleukin-1 **			X	X		X	X
C-reactive protein **			X	X		X	X
Palatability survey			5 weeks (AID-IC)			5 weeks (AID-IC)	
Dietary Inflammation Index							X
Focus group findings							X

* Genitourinary Pain Index (GUPI), Female Sexual Functioning Inventory (FSFI), and RAND Interstitial Cystitis Epidemiology Study (RICE) Bladder Symptom Impact Scale. ** Blood serum inflammatory biomarker.

**Table 3:** Participant demographics, comorbid conditions, and trigger foods.

Demographics, Comorbid Conditions, Trigger Foods	No.	%
Race/Ethnicity		
White/Non-Hispanic	10	100
Age Range		
25 to 34 years	1	10
35 to 44 years	1	10
45 to 54 years	4	40
55 to 64 years	1	10
64 to 75 years	3	30
Annual Income		
Prefer not to answer	1	10
USD 35,000 to USD 49,999	1	10
USD 50,000 to USD 74,499	3	30
USD 75,000 to USD 99,999	1	10
Over USD 100,000	4	40
Comorbid Conditions		
Overweight/obesity	8	80
Hypertension	2	30
Other chronic pain condition	7	70
Trigger Foods		
Tomatoes/tomato products	6	60
Alcohol	5	50
Spicy Foods	5	50
Citrus Food	3	3
Coffee	3	3

**Table 4:** Descriptive statistics and results of paired t-tests of dietary nutrient composition.

	Pre-Control Diet		Post-Control Diet		*p* Value	Pre-Test Diet		Post-Test Diet		*p* Value
	Mean	Std. Dev.	Mean	Std. Dev.		Mean	Std. Dev.	Mean	Std. Dev.	
E-DII Score *	−0.63	1.44	−1.35	1.63	0.21	−1.61	1.53	−1.38	1.89	0.05
Energy (kcalories)	1796.29	809.06	1757.80	639.76	0.91	1736.31	675.05	1578.08	715.07	0.62
Protein (g)	78.14	30.10	79.59	19.08	0.90	79.31	26.38	83.29	41.95	0.80
Total Fat (g)	0.55	0.80	0.34	0.54	0.49	0.55	1.02	0.39	0.72	0.70
MUFA (g) **	22.28	11.08	23.36	10.75	0.83	23.36	16.40	21.27	10.04	0.74
PUFA (g) **	27.22	14.84	27.23	11.32	0.99	28.46	11.70	26.52	20.57	0.80
Solid Fats (g)	26.90	18.41	25.33	16.37	0.84	26.89	25.46	22.33	16.94	0.64
Saturated Fats (g)	258.28	93.90	290.57	114.74	0.50	253.89	115.93	256.55	78.89	0.10
Cholesterol (mg)	184.28	60.90	245.18	223.21	0.42	295.95	127.58	264.75	206.40	0.69
Carbohydrates (g)	206.13	106.54	196.56	89.89	0.83	191.77	87.05	168.72	84.36	0.56
Fiber (g)	22.55	8.43	18.11	7.09	0.22	21.46	4.46	20.30	7.19	0.67
Refined Grains (oz)	3.25	2.45	3.87	2.38	0.57	2.05	1.86	2.38	2.28	0.73
Beta-carotene (mcg)	7457.66	9168.47	4761.02	4634.70	0.66	3411.50	2044.78	5446.77	3281.86	0.40
Alpha carotene (mcg)	979.49	1021.02	678.60	1033.62	0.45	444.87	587.42	461.78	876.36	0.11
Beta-cryptoxanthin (mcg)	115.63	68.72	65.02	58.28	0.42	112.90	111.79	53.64	51.05	0.96
Thiamin (mg)	1.21	0.45	1.28	0.50	0.52	1.22	0.44	1.25	0.59	0.88
Riboflavin (mg)	1.59	0.62	1.68	0.53	0.09	1.92	0.84	1.96	0.95	0.91
Niacin (mg)	21.08	8.73	20.24	4.92	0.74	22.02	7.95	20.86	15.21	0.83
Vitamin B6 (mg)	1.82	0.49	1.73	0.60	0.72	2.21	0.36	2.02	0.89	0.54
Vitamin B12 (mcg)	3.61	1.74	3.95	2.12	0.80	4.55	1.96	5.20	2.95	0.57
Vitamin C (mg)	85.82	41.42	64.33	35.36	0.70	113.83	59.07	60.69	31.67	0.02
Vitamin D (mg)	19.30	7.44	18.99	10.03	0.70	19.19	5.69	13.99	6.28	0.07
Vitamin E (mg)	0.77	1.33	0.04	0.14	0.23	0.21	0.65	0.00	0.00	0.33
Folic acid (mcg)	335.80	73.28	361.63	130.46	0.94	388.88	115.09	420.02	227.52	0.70
Iron (mg)	10.78	3.42	13.76	4.47	0.10	11.24	3.87	14.59	9.27	0.31
Magnesium (mg)	315.78	121.63	298.49	113.52	0.60	333.82	86.85	316.31	171.70	0.78
Selenium (mcg)	110.57	35.83	107.77	29.56	0.11	108.10	44.64	120.05	90.48	0.71
Zinc (mg)	9.44	3.62	11.98	4.44	0.75	11.12	4.40	10.69	6.79	0.87

* E-Dietary Inflammation Index (E-DII): scale −1 to +1 (−1 = strongly anti-inflammatory, 0 = no effect, and +1 = strongly pro-inflammatory). ** MUFA = monounsaturated fats, PUFA = polyunsaturated fats.

**Table 5:** Descriptive statistics and results of paired T-tests of disease activity measures.

Timepoint		N *	Mean	Std. Dev.	*p*-Value
Therapeutic Diet	RICE **	9	11.33	8.22	0.05
	GUPI, ** Pain	9	18.33	2.30	0.31
	GUPI, Urinary symptoms	9	3.83	2.70	0.09
	GUPI, Quality of life	9	7.33	2.64	0.28
	GUPI, total	9	7.33	2.64	0.28
	FSFI **	9	3.11	2.06	0.40
Control Diet	RICE	9	12.56	6.18	0.06
	GUPI, Pain	9	19.17	1.60	0.40
	GUPI, Urinary symptoms	9	4.33	2.63	0.23
	GUPI, Quality of life	9	8.61	3.12	0.23
	GUPI, total	9	8.61	3.12	0.23
	FSFI	9	1.44	1.87	0.26

* 10 participants completed, 1 record was incomplete and, thus, it was excluded from this analysis. ** RAND Interstitial Cystitis (RICE): scale 0 to 35; scale for each question 0 to 7; 0 is no effect, 1 is a very small negative or bad effect, 7 is a very large negative or bad effect; Genitourinary Pain Index (GUPI): scale 0 to 45; pain subscale 0 to 23, urinary subscale 0 to 10; quality of life subscale 0 to 12; Female Sexual Function Index (FSFI): scale 0 to 36.
